# Behavioural Effects of Adult Vitamin D Deficiency in BALB/c Mice Are not Associated with Proliferation or Survival of Neurons in the Adult Hippocampus

**DOI:** 10.1371/journal.pone.0152328

**Published:** 2016-04-04

**Authors:** Natalie J. Groves, DanaKai Bradford, Robert K. P. Sullivan, Kyna-Anne Conn, Rasha Fahad Aljelaify, John J. McGrath, Thomas H. J. Burne

**Affiliations:** 1 Queensland Brain Institute, The University of Queensland, St Lucia, Queensland, Australia; 2 Queensland Centre for Mental Health Research, The Park Centre for Mental Health, Richlands, Queensland, Australia; 3 Discipline of Psychiatry, The University of Queensland, St Lucia, Queensland, Australia; 4 Commonwealth Scientific and Industrial Research Organisation, Queensland Centre for Advanced Technologies, Pullenvale, Queensland, Australia; Centre national de la recherche scientifique, University of Bordeaux, FRANCE

## Abstract

Epidemiological studies have shown that up to one third of adults have insufficient levels of vitamin D and there is an association between low vitamin D concentrations and adverse brain outcomes, such as depression. Vitamin D has been shown to be involved in processes associated with neurogenesis during development. Therefore, the aim of this study was to test the hypothesis that adult vitamin D (AVD) deficiency in BALB/c mice was associated with (a) adult hippocampal neurogenesis at baseline, b) following 6 weeks of voluntary wheel running and (c) a depressive-like phenotype on the forced swim test (FST), which may be linked to alterations in hippocampal neurogenesis. We assessed proliferation and survival of adult born hippocampal neurons by counting the number of cells positive for Ki67 and doublecortin (DCX), and incorporation of 5-Bromo-2’-Deoxyuridine (BrdU) within newly born mature neurons using immunohistochemistry. There were no significant effects of diet on number of Ki67^+^, DCX^+^ or BrdU^+^ cells in the dentate gyrus. All mice showed significantly increased number of Ki67^+^ cells and BrdU incorporation, and decreased immobility time in the FST, after voluntary wheel running. A significant correlation was found in control mice between immobility time in the FST and level of hippocampal neurogenesis, however, no such correlation was found for AVD-deficient mice. We conclude that AVD deficiency was not associated with impaired proliferation or survival of adult born neurons in BALB/c mice and that the impact on rodent behaviour may not be due to altered neurogenesis per se, but to altered function of new hippocampal neurons or processes independent of adult neurogenesis.

## Introduction

Vitamin D deficiency is prevalent throughout the world [1], even in the temperate climate of Australia, approximately one in three people have insufficient levels (concentrations of <50 nmol/L of 25 hydroxyvitamin D) [2]. Vitamin D has well-known roles in calcium metabolism and bone health, in the immune system [3] and in brain development [4]. It is now thought that low vitamin D during adulthood may also be linked to adverse brain-related outcomes, such as cognitive impairment and depression in adults [5, 6]; as well as in neuropsychiatric and neurodegenerative diseases [4, 7].

A large population-based study showed that depression status and severity was associated with both decreased serum vitamin D and increased serum parathyroid hormone in older individuals [8]. In addition, a recent clinical trial was undertaken in adults suffering from depression who were also vitamin D deficient. Participants were given either a single dose of 150,000 IU or 300,000 IU vitamin D or no treatment and were tested again three months later. The single dose of 300,000 IU vitamin D not only proved safe but was also associated with a significant improvement in depression [9]. It has been hypothesized that hippocampal neurogenesis may play a role in the etiology of depression, although overall studies have shown complex and inconsistent results, as reviewed by Miller and Hen [10]. It also remains unclear if this process mediates the link between vitamin D status and brain outcomes.

Vitamin D is known to be anti-proliferative and have pro-differentiation effects, as shown by the addition of 1,25-dihydroxyvitamin D_3_ (1,25-(OH)_2_D_3_) to cultures of normal and malignant cell lines [11, 12]. Moreover, vitamin D has been shown to regulate a variety of neurotrophic factors, including nerve growth factor (NGF), providing further evidence for its ability to influence neuronal proliferation, differentiation, survival and growth [13–16]. Furthermore, the addition of 1,25-(OH)_2_D_3_ to neonatal subventricular zone cultures reduced neurosphere formation [17], once again supporting the anti-proliferative nature of vitamin D *in vitro*.

Conversely, vitamin D deficiency during gestation in Sprague-Dawley rats led to increased cell proliferation in embryos via altered gene expression of many cell cycle genes [18]. Similarly, the absence of vitamin D during gestation led to greater proliferation of neuroprogenitor cells, as seen by increased neurosphere production from cells dissociated from neonatal rat subventricular zone [17], providing further evidence from an animal model that the absence of vitamin D up-regulates proliferation during development. These studies were based on a developmental model of vitamin D deficiency (i.e. the rodents were exposed to low vitamin D only during gestation and early life). There is a paucity of information relating to neurogenesis and adult vitamin D deficiency.

A 1α-OHase knockout mouse that lacks the ability to make 1,25-(OH)_2_D_3,_ the active form of vitamin D, has been used to assess the effects of 1,25-(OH)_2_D_3_ deficiency on various aspects of adult hippocampal neurogenesis including proliferation and differentiation [19]. In the 1α-OHase knockout, hippocampal neurogenesis was no different to their wild-type or heterozygote littermates at three weeks of age [19]. However, the complete absence of 1,25-(OH)_2_D_3_ by eight weeks of age was associated with enhanced proliferation of progenitor cells in the dentate gyrus and a decrease in the survival of newborn cells without affecting differentiation [19]. This study suggested that the absence of vitamin D may be linked to the regulation of adult hippocampal neurogenesis by increasing both proliferation and cell death, which may or may not result in a net effect.

Taken together, the above studies suggest that vitamin D plays a role in proliferation and survival of new born neurons during gestation. The knockout mice suggest that there are effects of vitamin D post gestation, however, there is very little other data on the effects on adult neurogenesis in a range of mouse models. In order to explore the role of vitamin D in older BALB/c mice, independent of gestational effects, we sought to induce vitamin D deficiency through diet.

Markers for adult neurogenesis and cell proliferation include Ki67, doublecortin (DCX) and 5-Bromo-2’-Deoxyuridine (BrdU). Ki67 protein, an endogenous protein only present during active phases of the cell cycle, allows the assessment of cell proliferation. DCX is used as a marker for post-mitotic immature neurons and is an indicator of adult neurogenesis. A widely used molecule that marks dividing cells by integrating into DNA during mitosis, BrdU, is also a useful marker of cell proliferation [20]. BrdU can be co-localised with a neuron-specific nuclear protein, neuronal nuclei (NeuN), which is specific for mature neurons and expressed after terminal differentiation. Co-localisation of BrdU with NeuN marks newly generated neurons produced during BrdU administration [21] that have physically incorporated into the hippocampus.

Voluntary wheel running is robustly associated with increased hippocampal neurogenesis in adult mice [22, 23] and therefore is widely used to stimulate this process above low baseline rates. For example, eight-week old BALB/cByJ mice with access to running wheels for 43 days had a 450% increase in BrdU^+^ NeuN^+^ neurons in the dentate gyrus compared to sedentary mice [22]. Although this protocol does not capture rates of proliferation, assessing the number of surviving newborn neurons is a functional readout of the survival of newborn neurons into the hippocampus.

In combination, the assessment of cell proliferation, new neurons and survival of mature neurons in an enhanced neurogenesis model should allow detection of any impact of AVD deficiency on these aspects of adult hippocampal neurogenesis in BALB/c mice.

The forced swim test (FST) measures behavioural despair in rodents and is a well established behavioural assay related to the assessment of antidepressant agents [24]. We previously reported that AVD deficiency did not affect FST performance in group-housed male BALB/c mice [25]. However, BALB/c mice are a highly anxious strain and all mice spent a significant time immobile. It was proposed that there may have been a ceiling effect that prevented an effect of AVD deficiency from being apparent [25]. In addition to stimulating neurogenesis, voluntary wheel running in mice has been shown to reduce immobility in both the FST and tail suspension tests [26, 27]. To our knowledge, the effect of voluntary wheel running on immobility time in the FST has not been previously investigated in BALB/c mice. Impact of AVD deficiency on immobility time in the FST may indicate alterations in hippocampal neurogenesis.

The aims of this study were to test the effects of AVD deficiency on hippocampal neurogenesis. In line with studies in knockout mice [19], our primary hypothesis was that AVD deficiency would increase proliferation and decrease survival of adult born hippocampal neurons and that the increase in hippocampal neurogenesis stimulated by wheel running would be blunted by decrease in survival due to AVD deficiency. We aimed to test this hypothesis by assessing the number of Ki67^+^ cells as a marker of cell proliferation and DCX as a marker of immature neurons following behavioural tests. The number of BrdU^+^ NeuN^+^ cells was also examined as a measure of the number of newborn mature neurons surviving in the dentate gyrus at baseline and following voluntary wheel running. Secondly, we hypothesised that AVD-deficient mice would demonstrate behavioural correlates of AVD deficiency, specifically, spending *more time immobile* compared to controls in the FST, and this would correlate with altered neurogenesis as measured by proliferation and survival. We tested this by exploring the effect of wheel running on immobility time in the FST, and by assessing the correlation between neurogenesis and immobility time.

## Materials and Methods

### Animals and housing

A total of 101 BALB/c mice (71 male and 30 female) were used in this study. Ten-week old BALB/c mice (Animal Resources Centre, Canning Vale, WA, Australia) were obtained and housed in groups of four animals in individually ventilated OptiMICE cages, with bedding and nesting material at the Queensland Brain Institute Animal House Facility, The University of Queensland, Australia.

The mice were assigned to either a control diet (Standard AIN93G Rodent diet with 1,500 IU vitamin D_3_/kg (prior to irradiation with 25 kGy), Specialty Feeds, WA, Australia) or a vitamin D-deficient diet (Vitamin D Deficient AIN93G Rodent diet with 0 I.U vitamin D_3_/kg irradiated with 25 kGy, Specialty Feeds, WA, Australia) for 10 weeks prior to the start of behavioural testing and for the entire duration of the experimental procedures. The mice were maintained on a 12-hour light-dark cycle (lights on at 07:00 h) with *ad libitum* access to food and water. They were housed under incandescent lighting free from UVB radiation. All experimental work was performed with approval from The University of Queensland Animal Ethics Committee, under the guidelines of the National Health and Medical Research Council of Australia.

### Experimental Design

After 10 weeks on the diet, the first wave of mice were separated into the following groups. Twenty-eight mice (*n* = 5–8/sex/group) were individually housed with running wheels and 28 mice (*n* = 6–9/sex/group) were individually housed without running wheels, for the next six weeks. An additional four mice (2 male and 2 female) were individually housed without running wheels as non-BrdU injected controls. These mice were not included in the behavioural analysis but were used as a negative control to ensure that the only BrdU analysed was from the injections of BrdU. Mice were placed into the following five groups: Control Runner, Control Non-runner, AVD-deficient Runner, AVD-deficient Non-runner and non-BrdU injected control.

Wheel rotations for running groups were collected in 60 min time bins over the 6-week period using automated Wheel Manager software. During the first 10 days of individual housing all mice, excluding the non-BrdU controls, received a daily i.p injection of 50 mg/kg of BrdU at the same time each day, between 1000–1200 h, to assess hippocampal neurogenesis at the end of the behavioural testing. After 16 weeks on the diet, and at the end of running wheel access for the Running groups, mice were tested in a 30 min activity monitor test and in the FST. See Section on behavioural testing below for full details of these methods. These tests were chosen as they are altered by wheel running [27] and the forced swim test was included as a measure of behavioural despair.

The second wave of mice included 27 male mice, 14 mice (*n* = 6–8/group) were individually housed with running wheels and 13 mice (*n* = 6–7/group) were individually housed without running wheels, for the next six weeks. These mice were not injected with BrdU, but were analysed for serum vitamin D levels after 16 weeks on the diet.

The third wave of mice (*n* = 14, male, 7 per group), were subjected to diet conditions and underwent analysis for DCX protein expression.

### Serum 25-hydroxyvitamin D_3_

A terminal blood sample was taken from each mouse from the second wave via cardiac puncture. The levels of 25(OH)D_3_ was measured in serum samples using liquid chromatography-tandem mass spectrometry on a 4000 QTrap API AB mass spectrometer [28].

### Behavioural Testing

#### Activity monitor

Activity monitors were used to measure baseline locomotion [29]. Eight novel open field activity monitors were used. The arenas were 27.5 x 27.5 x 30 cm high and made of clear Perspex with two arrays of 16 x 16 infrared beams. One array of beams was low (1 cm above floor) so that beams were broken when the animal was present or moving, and another high (8 cm above floor) so beams were broken only when the animal reared. Mice were placed in the centre of the arena and their activity was monitored for 30 min, in which time the Med Associates software used the beam break data to triangulate the position of the mouse and measure the distance travelled by the mouse over time.

#### Forced swim test

The apparatus used in the forced swim test was a clear round container (20 cm high x 14 cm diameter) with a column of water (16 cm deep) maintained at 25°C. Each mouse was placed in the container for six min and recorded using a USB digital camera and recording software. Activity was scored using the mobility threshold settings within the Ethovision software by measuring the percentage change in area of the tracked object from one sample to the next. Immobility was defined as less than 5% movement using these settings and was validated by a human observer.

### Immunohistochemistry

To ensure our results were comparable with previous research on exercise induced hippocampal neurogenesis, immunohistochemistry was conducted following an established protocol [22].

#### Perfusions

All mice were anaesthetized by an i.p injection of Lethabarb at 100 mg/kg body weight 24 h after FST and transcardially perfused with 40 ml of phosphate buffered saline (PBS) and 40 ml of 4% paraformaldehyde (PFA) in 0.1 M PBS. The brains were removed and post fixed in 4% PFA for 24 h before being stored in sodium azide (0.05%) in 0.1M PBS (PBS azide). BrdU incorporation was assessed in *n* = 8 per group. Brains included in the neurogenesis analysis were selected blind to behavioural results. Brains were transferred to a 30% sucrose solution for 24 h and then rapidly frozen and 50 μm sections (40 μm for the third wave) containing the hippocampus were cut coronally using a sledge microtome and stored in PBS azide at 4°C until used.

#### Double-labeled immunofluorescence

A one-in-five series was stained to identify proliferating and newly divided mature neurons. Sections were rinsed with PBS azide to remove all trace of sucrose. Sections were incubated in 1M HCl for 20 min at 40°C to denature DNA, then rinsed with 0.1M Boric acid for five min. The sections were then incubated in Antigen Recovery Solution for 30 min at 60°C, followed by 3x5 min rinses with PBS azide. Sections were blocked at RT with a solution of 3% normal goat serum (NGS) and 0.05% Saponin in PBS azide. Sections were incubated with the primary antibodies; rat anti-BrdU (1:500) (Accurate Chemical, NY, USA) and mouse anti-NeuN (1:500) (Merck, Millipore, MA, USA), or with the primary antibody, rabbit anti-Ki67 (1:5000) (Abcam, Cambridge, UK) for 56 h at 4°C. Sections were then washed for 3x15 min in PBS azide. Secondary antibodies raised in donkey or goat were conjugated with fluorescent markers (Alexa fluor-488 anti-rat (1:2000) and Alexa fluor-647 anti-mouse (1:500) (Jackson Lab. Inc, PA, USA), and Alexa fluor-568 anti-rabbit (1:1000) (Thermo Fisher, IL, USA) and the sections were incubated with the secondary antibodies overnight at 4°C. Sections were then washed with 1:1000 DAPI in PBS azide for 15 min, followed by 2x15 washes in PBS azide. Sections were mounted from a solution of chrome alum gelatin onto slides with DABCO.

To assess the number of immature neurons in the third wave of mice, a one-in-six series was labelled. Sections were washed 3x10 min with PBS and blocked with a solution of 2% NGS with 0.3% Triton in PBS for 1 hr. After washing 3x10 min PBS, sections were incubated overnight at room temperature with the primary antibody; rabbit anti-Dcx AB18723 (1:1000) (Abcam, Cambridge, UK). Sections were again washed 3x10 min with PBS. Secondary antibody (conjugated with fluorescent markers), goat anti- Rabbit 488 (1:1000) was added with DAPI (1:1000) 2 hours at room temperature. The sections received a final wash 3x10min with PBS before being mounted with ProLong Gold (Thermo Fisher, IL, USA).

#### Image analysis

**Fig 1:**
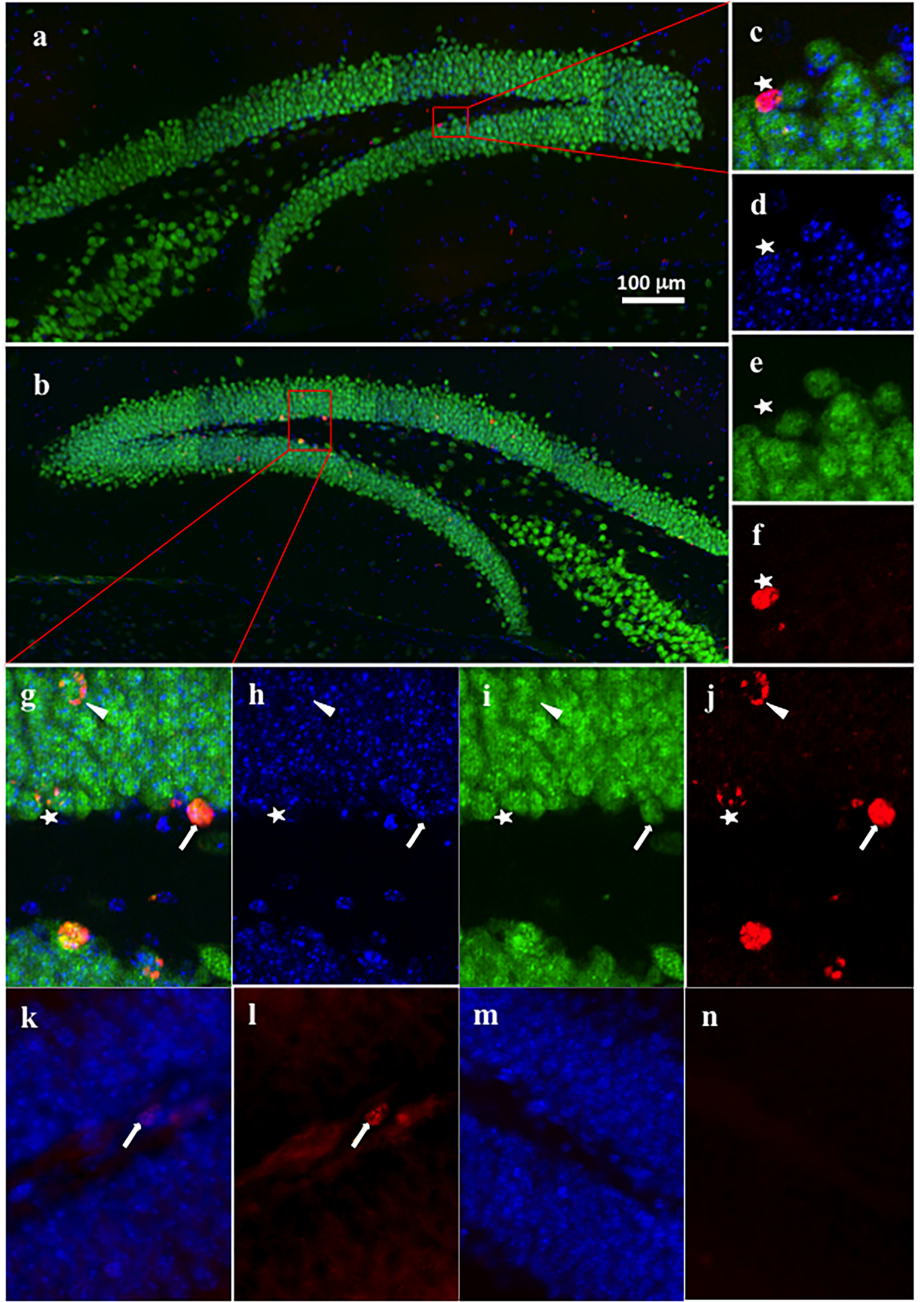
Representative images of BrdU incorporation within mature neurons in the dentate gyrus. A representative image of the dentate gyrus from a Non-Runner **(a)** and a Runner **(b).** A close up image from the section of the dentate gyrus from the Non-Runner **(c),** and the Runner **(g),** DAPI stain showing nuclei **(d and h)**, NeuN stain showing mature neurons **(e and i)** and BrdU staining showing newly born cells **(f and j)**. The line arrow points to a mature neuron (stained with NeuN) with complete BrdU incorporation, these cells have not undergone cell division after the uptake of BrdU. The arrow head points to a mature neuron (stained with NeuN) with only partial BrdU incorporation, these cells have divided following BrdU injection or were born late in the accumulation window. The star points to a cell with BrdU incorporation but no co-localisation with NeuN and is therefore not a neuron (not analysed). Blue–DAPI, Green–NeuN, Red–BrdU. A representative image of Ki67 staining (arrow, k and l) and control (m and n). Blue–DAPI, Red–Ki67.

Fluorescence images were captured on a Zeiss Axio Imager Z1 microscope using a 20x 0.8NA PlanApo objective and Zeiss AxioCam HRm camera. Optical sectioning was achieved using ApoTome structured illumination and the large area of the dentate gyrus was captured using the tiled imaging and stitching features of Zen 2009 software. Cells that exhibited complete incorporation of BrdU and those that exhibited partial accumulation of BrdU were all counted as BrdU^+^ NeuN^+^ cells. Cells with nuclei completely incorporated with BrdU and colabeled with NeuN, have not undergone any divisions. Partially immunolabelled cells, where nuclei was stained with NeuN but BrdU was only partially incorporated, have either undergone division after BrdU was injected into the animals (daughter cells) or were born late in the accumulation window **(Fig 1)**. For Ki67 immunoreactivity, only Ki67^+^ cells were included in the analysis. All DCX^+^ cells in the dentate gyrus were counted.

### Statistical analysis

Data were analysed using SPSS version 20. All data were analysed for the main effects of Diet (control or AVD-deficient) and Sex using analysis of variance (ANOVA), except the DCX data, which were analysed with Student’s *t* test. Repeated measures ANOVA was used for the wheel rotations, activity monitors and the forced swim test results. Significant differences (*p*<0.05) were followed up with post-hoc *t*-tests (Bonferroni), however there were no significant post-hoc *t*-test results. Correlations between neurogenesis (Ki67, BrdU counts) and immobility time were assessed using Pearson’s test for correlation. Adult hippocampal neurogenesis was measured as the total number of BrdU^+^ cells co-expressing NeuN averaged per section counted, the number of Ki67^+^ cells averaged per section counted, and the number of DCX^+^ cells per section counted, with the total area counted not different between groups.

## Results

### Vitamin D deficient diet significantly reduces serum vitamin D levels

To demonstrate the effectiveness of the vitamin D deficient diet, blood sera were analyzed. There was a main effect of Diet (*F*_1,23_ = 737.9, *p* < 0.001) on serum 25(OH)D_3_ levels but no significant effect of Running (*F*_1,23_ = 1.07, *p* = 0.313), with no interaction (*F*_1,23_ = 0.01, *p* = 0.940). Levels of 25(OH)D_3_ were significantly reduced in both AVD-deficient Runners (2.23 ± 0.23 nM) and AVD-deficient Non-runners (3.44 ± 0.67 nM) compared to both Control Runners (31.99 ± 0.70 nM) and Control Non-runners 33.03 ± 2.21 nM. As no diet by sex interactions were found, data from male and female mice were pooled.

### Running wheel activity is unaffected by AVD deficiency

It has been well documented that volitional exercise stimulates adult neurogenesis in mice [22]. In order to ensure propensity for wheel running did not contribute to differences between AVD-deficient and control mice, we compared mice individually housed with and without running wheels for six weeks (*n* = 28 gender and diet matched per group). There was no significant main effect of Diet (*F*_1,14_ = 1.24, *p* = 0.285) on the number of wheel rotations measured between Control (*Mean±SEM*; 15,388±1,215 rotations per day) and AVD-deficient (13,000±1,907 rotations per day) mice. Hence it appears that AVD deficiency did not impact on voluntary wheel running in BALB/c mice, and that any differences between the groups are not due to variations in volitional exercise.

### Volitional exercise, but not diet, affects spontaneous locomotion

**Fig 2:**
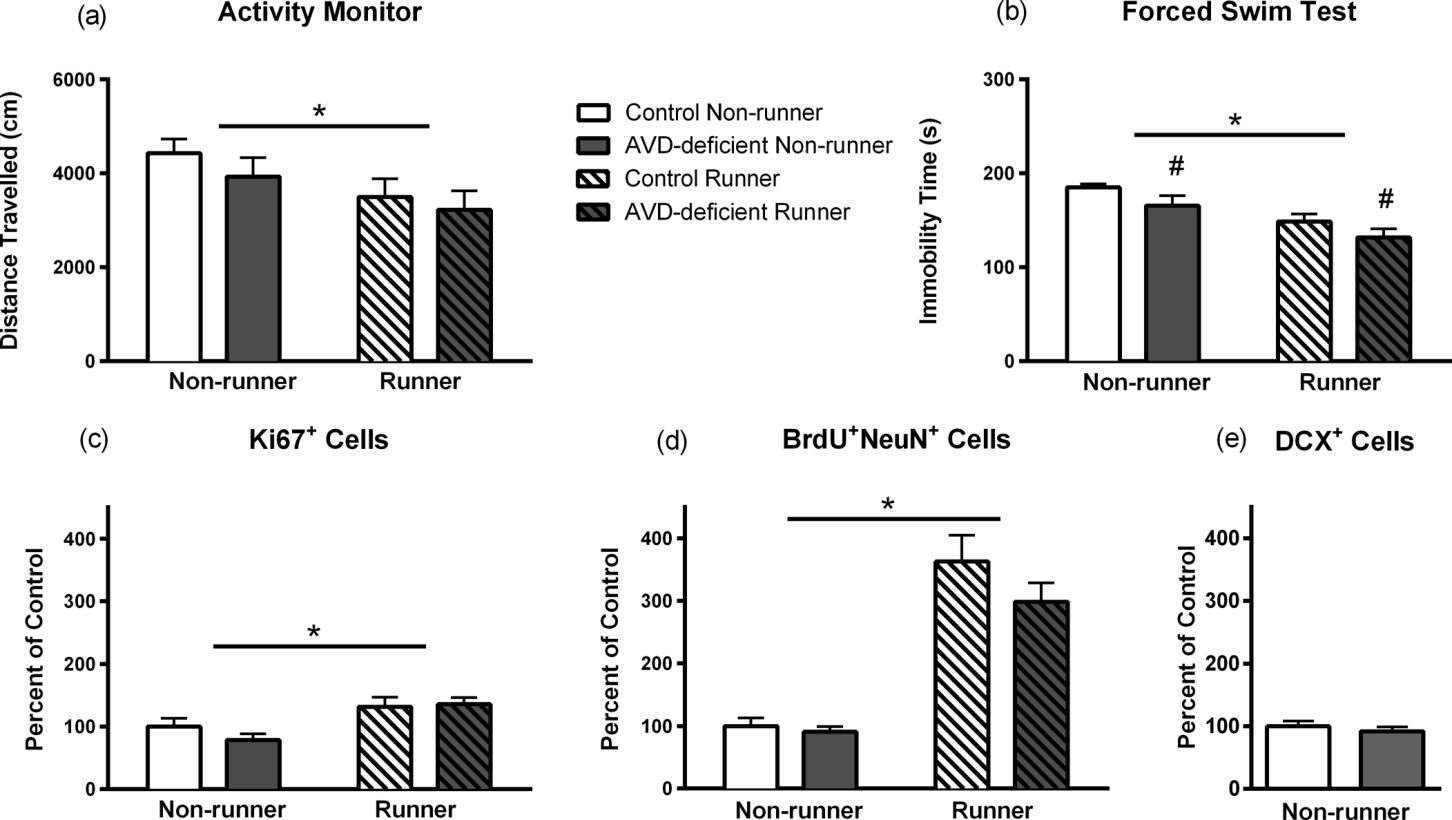
Results for the activity monitor, forced swim test (FST), and Ki67^+^, BrdU^+^ NeuN^+^, and DCX^+^ cell counts. The distance travelled in the activity monitors is shown in **(a)** (*n* = 13–15 per group). There was no significant effect of Diet but there was a significant main effect of Running. Runners moved less than Non-runners. Immobility time during the FST is shown in **(b)** (*n* = 7–8 per group). There was a main effect of both Diet and Running. Wheel running significantly reduced immobility time; moreover AVD deficiency also reduced immobility time. The number of Ki67^+^ is shown expressed as a percent of control non-runner values **(c)** (*n* = 8 per group). There was no significant effect of diet but there was a significant effect of Running. Runners had higher numbers of Ki67^+^ cells compared to Non-runners. There was no significant difference between control and AVD-deficient mice. BrdU^+^ NeuN^+^ cell counts expressed as a percent of control non-runner values is shown in **(d)** (*n* = 8 per group). There was a significant increase in the number of BrdU^+^ NeuN^+^ cells following voluntary running. However, there was no significant effect of Diet on baseline cell numbers or cell numbers stimulated by voluntary running. The number of DCX expressed as a percent of control non-runner values is shown in **(e)** (*n* = 7 per group). Where main effect is significant, p-value is given on graph. Mean ± SEM (* *p* < 0.05).

In an earlier study [25], we found significant differences in spontaneous locomotion between male AVD-deficient and control BALB/c mice in both novel and familiar open field tests. In the novel environment, locomotion was increased for AVD-deficient mice, however, in the familiar environment, activity was significantly decreased compared to controls. These findings could be explained by differences in handling, where in the novel environment the mice had never been handled, and were therefore likely to be more active. To further explore differences in locomotion we compared the mice housed with and without running wheels in novel open field activity monitors. In this study, there was no significant main effect of Diet (*F*_1,51_ = 1.03, *p* = 0.314) on the distance travelled in the activity arena. There was, however, a main effect of Running (*F*_1,51_ = 4.66, *p* = 0.036), where Non-runners were ~25% more active (*Mean±SEM*; 4431±299 cm control; 3932±407 cm AVD deficient) than Runners (3496±389 cm control; 3220±413 cm AVD deficient) with no interaction with diet (*F*_1,51_ = 0.85, *p* = 0.772, *n* = 13–15 per group, see **Fig 2A).** This effect was stronger for males than for females suggesting that males are more sensitive to the activity monitor testing.

### Volitional running reduces immobility time in the forced swim test, particularly for AVD-deficient mice

Our final behavioural test aimed to determine any differences in immobility time, a behavioural correlate of behavioural despair. There were significant main effects of Diet (*F*_1,27_ = 4.67, *p* = 0.040) and Running (*F*_1,27_ = 17.71, *p* < 0.001) on immobility time in the FST, with no interaction (*F*_1,27_ = 0.23, *p* = 0.879) (*n* = 7–8 per group). Mice exposed to running wheels had a significant reduction in immobility time (*Mean±SEM*; 148±8 s control; 131±109 s AVD deficient) compared to those without access to running wheels (185±3 s control; 165±10 s AVD deficient). This result is not unexpected; running is associated with an anti-depressant effect reflected in decreased immobility in the FST [26]. However, AVD-deficient mice spent less time immobile (148±8 s) compared to controls (166±10 s, see **Fig 2B)**. We expand on this in the discussion.

### Volitional exercise, but not diet, is reflected in cell proliferation and survival

While in utero vitamin D deficiency has been shown to lead to increased proliferation in rat neonatal neurospheres [17], there is some evidence to suggest this effect is limited to development [18]. To determine if AVD deficiency regulated proliferation in BALB/c mice, we looked at proliferation of cells in the dentate gyrus following the behavioural testing. There was no significant difference between all groups in the area (mm^2^) of the dentate gyrus in which Ki67^+^ cells were counted (*F*_1,25_ < 1.58, *p* > 0.220) (**Fig 1K**). While there was no significant main effect of Diet (*F*_1,25_ = 0.43, *p* = 0.517) on the number of Ki67^+^ cells per mm^2^ (*n* = 6–8 per group), we did see a significant main effect of Running (*F*_1,25_ = 11.29, *p* = 0.003) where Runners had a greater number of Ki67^+^ cells (*Mean±SEM*; 85±10 cells per mm^2^ control; 87±7 cells per mm^2^ AVD deficient) compared to Non-runners (64±9 cells per mm^2^ control; 51±6 cells per mm^2^ AVD deficient, see **Fig 2C)**. This suggests that, in BALB/c mice, AVD deficiency does not regulate proliferation, and nor does it impact processes that do regulate proliferation, such as volitional exercise.

To determine if AVD deficiency impacted neurogenesis in the adult hippocampus through mechanisms other than proliferation, we looked at the number of newborn neurons surviving in the dentate gyrus. There was no significant difference between all groups in the area (mm^2^) of the dentate gyrus in which BrdU^+^ NeuN^+^ cells were counted (*F*_1,28_ < 0.55, *p* > 0.464). In accord with previous research [22, 23], there was a significant main effect of Running (*F*_1,28_ = 74.85, *p* < 0.001) on the number of BrdU-incorporated cells per mm^2^, with Runners having a higher number of BrdU^+^ NeuN^+^ cells (*Mean±SEM*; 197±23 cells per mm^2^ control; 162±17 cells per mm^2^ AVD deficient) compared to Non-runners (54±7 cells per mm^2^ control; 49±5 cells per mm^2^ AVD deficient). There was, however, no significant main effect of Diet (*F*_1,28_ = 1.82, *p* = 0.188) on the number of BrdU-incorporated cells and there was no interaction (*F*_1,28_ = 1.00, *p* = 0.326, *n* = 8 per group, see **Fig 2D)**. These findings complement our proliferation results.

To further ensure that AVD deficiency did not impact on the early processes of hippocampal neurogenesis, we subjected a third wave of mice (*n* = 14, 7 per group), to the AVD-deficient diet conditions and analysed the number of immature neurons generated. Consistent with our proliferation and survival findings, there was no significant difference in the number of DCX+ cells counted in a one in six series in the dentate gyrus of AVD-deficient mice (*Mean±SEM*; 50±4 cells per mm^2^) compared to control mice (46±3 cells per mm^2^), (*t*_12_ = 0.074, *p* = 0.474) (**Fig 2E**).

### The number of surviving adult born neurons correlates with immobility time in the FST for control mice only

There was no significant correlation between Ki67^+^ cells and time spent immobile (R^2^ = -0.085, *p* = 0.747, N = 17). This finding held true when the groups were separated for Diet, with no significant correlation between Ki67^+^ cells and time spent immobile in Controls (R^2^ = 0.098, *p* = 0.788, *n* = 10) or AVD-deficient mice (R^2^ = -0.345, *p* = 0.449, *n* = 7). This finding suggests that immobility time is unrelated to proliferation of adult born cells in the dentate gyrus. The third wave of mice, in which DCX^+^ cells were counted, did not undergo FST testing.

Overall, there was no significant correlation between BrdU^+^ NeuN^+^ cells and time spent immobile (R^2^ = -0.416, *p* = 0.077, *n* = 19), however when the groups were separated, a significant correlation between BrdU^+^ NeuN^+^ cells and time spent immobile was found in control mice (R^2^ = -0.810, *p* = 0.005, *n* = 10), but not in AVD-deficient mice (R^2^ = -0.166, *p* = 0.670, *n* = 9). This suggests that while AVD deficiency does not impact on the number of dividing cells, immature cells or their differentiation into mature neurons; it may impact the function of newly generated cells.

## Discussion

The main finding from this study was that AVD deficiency did not affect proliferation or survival of adult hippocampal neurons within the dentate gyrus, at baseline or after voluntary wheel running in BALB/c mice. Nor did we see any difference in the number of immature neurons following behavioural testing. Therefore, we can reject the hypothesis that AVD deficiency impairs adult hippocampal neurogenesis through alterations in proliferation or survival into the dentate gyrus in BALB/c mice. As predicted, wheel running was associated with a significant increase in hippocampal neurogenesis in both male and female mice in this study, however this was not altered by AVD deficiency. Furthermore, there was no confounding factor from AVD deficiency altering the amount of voluntary wheel running and therefore confounding neurogenesis results, because voluntary wheel running was not significantly different between diets. Overall, this study provides convincing evidence that any putative link between AVD deficiency and adverse brain-related outcomes is not mediated via proliferation or survival of new hippocampal neurons in BALB/c mice as measured in this study.

Hippocampal neurogenesis was measured using well-known techniques, using a modified version of the protocol used in a previous study [22]. We measured the number of BrdU^+^ NeuN^+^ cells and Ki67^+^ cells within the dentate gyrus of the hippocampus, within a set number of sections from a one in five series. We further measured the number of DCX^+^ neurons in a one-in six series. This allowed us to measure three aspects of hippocampal neurogenesis, proliferating cells, immature cells and cells integrating into the dentate gyrus. Our counts are consistent across all three experiments and consistent with previous studies [22, 30]. We have clearly shown that both these processes were not impaired by AVD deficiency, furthermore, AVD deficiency had no effect on processes that enhance proliferation, such as wheel running.

One of the difficulties in unravelling the mechanisms by which AVD deficiency impacts rodent behaviour is the variation between strains, not just in response to AVD deficiency but in neurogenesis itself. A comparison of neurogenesis in four strains found that while differences in proliferation rates did not reach significance, number of new neurons, neuronal differentiation and survival all differed [31]. Similar genetic variations are seen following exercise, with the number of BrdU^+^ cells varying with genotype [22]. Consistent with our results, BALB/c mice were found to have a four-fold increase in BrdU^+^ cells following running [22].

The 1α-OHase knockout mouse is most relevant to our study due to the role of 1α-OHase in the conversion of vitamin D to 1,25(OH)_2_D_3_ and the BALB/c background.

These mice have an increase in proliferation and a decrease in the survival of newborn neurons in the hippocampus at eight weeks of age [19], yet we have shown that after 16 weeks on a vitamin D deficient diet, with serum levels of 25(OH)D_3_ known to be deplete after six weeks, there was no impairment of hippocampal proliferation or survival of new neurons. This shows that either the AVD model used in this study may be subtler than a knockout animal model and therefore more reflective of vitamin D deficiency in human populations, or alternatively, it may be the developmental aspect of the 1α-OHase knockout mouse that leads to hippocampal neurogenesis alterations in the adult animal.

Despite no change in cell proliferation or survival with AVD deficiency, there was a significant effect of diet on behaviour in the FST. We have previously shown that AVD deficiency did not impact immobility time on the FST in group-housed male BALB/c mice. However in this study, we chose to use individual housing to allow accurate measurement of individual wheel running in both male and female mice and we found that AVD deficiency resulted in a reduction in immobility time during the FST. A previous study in Swiss-Webster mice showed a significant difference in immobility time between individually housed mice and group-housed mice, showing that the FST was sensitive to housing conditions [32]. This study has showed that in individually housed BALB/c mice, wheel running for six weeks leads to a reduction in immobility time, and that this effect is enhanced for AVD-deficient mice.

We hypothesized that AVD deficiency would increase immobility compared to controls. Based on our results we can reject this hypothesis. AVD deficiency in BALB/c mice does not induce a depressive-like phenotype on the FST. These results are converse to those expected if vitamin D plays a role in depression, but are consistent with recent research demonstrating that immobility in the FST is independent of hippocampal neurogenesis in BALB/c mice [33].

There are a number of epidemiological studies that show a significant association between vitamin D deficiency and depression [6, 8] and there have also been clinical trials showing reductions in depressive symptoms following vitamin D supplementation [9] yet this study produced no depressive-like symptoms in the AVD-deficient BALB/c mice as measured by immobility time in the FST. As mentioned above, this could be due to strain, alternatively it is possible that the mice were not on the diet for a long enough period. While the AVD-deficient mice are deplete of serum 25(OH)D_3_ prior to the start of behavioural testing, it may require a longer period of depletion before some symptoms are detectable and this could be addressed in future studies.

Alternatively, it is possible that a secondary insult may be required to exacerbate the effects of vitamin D deficiency to induce depressive-like symptoms [7]. For example, many of the epidemiological studies that show an association between vitamin D deficiency and depression are performed in older adults [8]. A recent study performed in rats showed no effect of vitamin D supplementation in young (6 month old) rats, however in older rats (20 month old), vitamin D supplementation significantly improved learning and memory [34]. Another study showed that long-term vitamin D dietary manipulation in aging rats (11–13 months old at the start of the study and on diet for 5–6 months), had a significant impact on learning with only the rats on a high vitamin D diet able to perform a complex memory task, compared to those on low or mid levels [35]. Therefore, perhaps combining vitamin D deficiency with ageing or another secondary insult such as social stress, could lead to a depressive-like phenotype in BALB/c mice.

Similar to this study, a recent study in a transgenic Alzheimer’s Disease mouse model showed a reduction in immobility time during the FST and it was proposed that this result may be due to a lack of cognitive flexibility and coping with stress strategies rather than from a change in behavioural despair [36]. However, as determining the mechanism leading to the reduction in immobility time with AVD deficiency was beyond the scope of our experiments, future studies should include testing the cognitive abilities of AVD-deficient mice.

BrdU^+^ NeuN^+^ cells and Ki67^+^ cells were counted in a subset of the population that also underwent the FST. Post-hoc analysis showed that immobility time was not significantly correlated with the level of proliferation. Interestingly, however, a significant negative correlation was found between BrdU^+^ NeuN^+^ cells and immobility time for control mice only, where a greater number of cells was correlated with reduced immobility. This effect was not seen for AVD-deficient mice, potentially suggesting that the role of new neurons differs in the AVD-deficient model. Previous studies have shown that in C57BL/6 mice, hippocampal neurogenesis is required for the beneficial effects of antidepressants in the FST (reduction in immobility time) [37]. In BALB/c mice, however, ablation of neurogenesis does not prevent the reduction in immobility time seen with chronic antidepressant treatment [33]. Taken together, the results from [33] and the results from this study may suggest that immobility time in the FST is independent of changes in neurogenesis in BALB/c mice and that there are other mechanisms involved.

While this study has shown that the number of proliferating cells, number of immature cells and the number of newborn neurons surviving in the hippocampus were not affected by AVD deficiency, there are other measures of interest that may be impacted by AVD deficiency, such as the extent of branching of immature neurons, and level of apoptosis, differentiation, and protein expression [18, 19, 35] which should be investigated in future studies.

This is the first study to show that AVD deficiency did not impair proliferation or survival of adult hippocampal neurons at baseline or after voluntary wheel running, despite a behavioural alteration on the FST. This suggests that the behavioural effects of AVD deficiency are not mediated via early hippocampal neurogenesis processes and that other mechanisms may be involved. In light of the fact that there is growing evidence to support the hypothesis that vitamin D deficiency in adulthood is related to adverse brain outcomes, it is now important to unravel the mechanisms by which vitamin D deficiency alters brain function.

## Supporting Information

S1 File Raw Data A Activity Monitors in S1 File. Raw Data B Forced Swim Test in S1 File. Raw Data C Ki67 Cell Counts in S1 File. Raw Data D Neurogenesis in S1 File. Raw Data E Running Wheels in S1 File. Raw Data F Serum vitamin D in S1 File. Raw Data G DCX Cell counts in S1 File.
(ZIP)
